# A Comparison of Hematological, Immunological, and Stress Responses to Capture and Transport in Wild White Rhinoceros Bulls (*Ceratotherium simum simum*) Supplemented With Azaperone or Midazolam

**DOI:** 10.3389/fvets.2020.569576

**Published:** 2020-10-20

**Authors:** Friederike Pohlin, Emma H. Hooijberg, Peter Buss, Nikolaus Huber, Francois P. Viljoen, Dee Blackhurst, Leith C. R. Meyer

**Affiliations:** ^1^Centre for Veterinary Wildlife Studies, Faculty of Veterinary Science, University of Pretoria, Pretoria, South Africa; ^2^Department of Interdisciplinary Life Sciences, Research Institute of Wildlife Ecology, University of Veterinary Medicine, Vienna, Austria; ^3^Department of Paraclinical Sciences, Faculty of Veterinary Science, University of Pretoria, Pretoria, South Africa; ^4^Department of Companion Animal Clinical Studies, Faculty of Veterinary Science, University of Pretoria, Pretoria, South Africa; ^5^Veterinary Wildlife Services, Kruger National Park, South African National Parks, Skukuza, South Africa; ^6^Pharmacology, School of Pharmacy and Centre of Excellence for Pharmaceutical Sciences, Faculty of Health Sciences, North-West University, Potchefstroom, South Africa; ^7^Division of Chemical Pathology, Faculty of Health Sciences, University of Cape Town, Cape Town, South Africa

**Keywords:** acute phase response, epinephrine, hemoconcentration, leukocyte coping capacity, N:L ratio, oxidative stress, translocation, wildlife

## Abstract

Capture and transport are essential procedures for the management and conservation of southern white rhinoceroses (*Ceratotherium simum simum*), but are associated with stress-induced morbidity and mortality. To improve conservation efforts, it is crucial to understand the pathophysiology of rhinoceros stress responses and investigate drug combinations that could reduce these responses. In this study we measured rhinoceros stress responses to capture and transport by quantifying hematological and immunological changes together with adrenal hormone concentrations. We investigated whether the potent anxiolytic drug midazolam was able to mitigate these responses compared to azaperone, which is more commonly used during rhinoceros transport. Twenty three wild white rhinoceros bulls were transported for 6 h (280 km) within the Kruger National Park for reasons unrelated to this study. Rhinoceroses were immobilized with either etorphine-azaperone (group A, *n* = 11) or etorphine-midazolam (group M, *n* = 12) intramuscularly by darting from a helicopter. Azaperone (group A) or midazolam (group M) were re-administered intramuscularly every 2 h during transport. Serial blood samples were collected at capture (TC), the start of transport (T0) and after 6 h of transport (T6). Changes in hematological and immunological variables over time and between groups were compared using general mixed models. Increases in plasma epinephrine and serum cortisol concentrations indicated that rhinoceroses mounted a stress response to capture and transport. Packed cell volume decreased from TC to T6 indicating that stress hemoconcentration occurred at TC. Neutrophils progressively increased and lymphocytes and eosinophils progressively decreased from T0 to T6, resulting in an increase in neutrophil to lymphocyte ratio; a characteristic leukocyte response to circulating glucocorticoids. A reduction in serum iron concentrations may suggest the mounting of an acute phase response. Rhinoceroses experienced a decrease in unsaturated fatty acids and an increase in lipid peroxidation products at capture and toward the end of transport indicating oxidative stress. Midazolam, at the dose used in this study, was not able to mitigate adrenal responses to stress and appeared to directly influence leukocyte responses.

## Introduction

Translocation is the deliberate human-mediated movement of individuals or populations of wild animals from one location to another (1). Hundreds of white rhinoceroses (*Ceratotherium simum*) are translocated each year for conservation purposes (2). Despite the widespread use and importance of this practice, rhinoceros translocations often result in morbidity and even mortality (3, 4). The current mortality rate for rhinoceros translocations in South Africa and Namibia is estimated to be 5% (2). Although the direct causes for these mortalities are often related to external factors, such as novel pathogens, vulnerability to these factors is likely exacerbated by hematological and immunological changes induced by a stress response to translocation (5, 6).

The term stress is an ambiguous concept in biology and biomedicine and is often defined as a threat to homeostasis (7). A more integrated definition states that stress is a constellation of events, consisting of an unexpected stimulus (stressor), that precipitates a reaction in the brain (stress perception), which activates physiological systems in response (stress response) (8, 9). The two most frequently studied physiological systems orchestrating the stress response are the autonomic nervous system (ANS) and the hypothalamic-pituitary-adrenal (HPA) axis (8). The response of the ANS to a stressor results in an almost immediate (milliseconds) increase in the release of the catecholamine neurohormone epinephrine from the adrenal medulla (10). Stimulation of the HPA axis results in a slower (minutes) but more sustained release of the glucocorticoid steroid hormone cortisol (in mammals) from the adrenal cortex (10).

These hormones induce cellular changes in various tissues and organs, provide information about the presence of a stressor, and also have significant effects on immune cell distribution and function (9). Specifically, these latter effects include hematological responses such as a decrease in lymphocytes and eosinophils and an increase in neutrophils (11), and immunological responses such as the mounting of an acute phase reaction (12) and oxidative stress (13). Because neutrophil and lymphocyte counts are affected by stress in opposite directions, the relative proportion of neutrophils to lymphocytes (N:L ratio) is frequently used as measure of a stress response in mammals and birds (14). In healthy individuals, leukocytes (particularly neutrophils) release reactive oxygen species (ROS) as an early cellular innate immune response against invading pathogens. The so-called leukocyte coping capacity (LCC) quantifies neutrophil ROS production in real time and is substantially reduced in stressed individuals (15). Thus, LCC has been applied in several mammalian and avian wildlife species as a quantitative indicator of stress and animal welfare during capture and handling (16, 17).

By inducing proinflammatory cytokines in immunity-related cells, the activation of the HPA axis also promotes the initiation of an acute phase reaction (18). The acute phase reaction represents a complex systemic reaction of the innate immune system to non-specific stimuli characterized by an increase in positive acute phase reactants (APRs) and decrease in negative APRs accompanied by the generation of ROS (12, 19). Oxidative stress is the result of an imbalance between the production of ROS and the endogenous antioxidant mechanisms, which counteract the effects of ROS (20). Unsaturated fatty acids, which are components of phospholipids and triglycerides, are particularly vulnerable to oxidation by ROS, leading to a process known as lipid peroxidation (20, 21). During lipid peroxidation, a hydrogen ion is removed from the unsaturated fatty acid and the remaining lipid radical undergoes molecular rearrangement to form a conjugated diene (CD) (20). Following a complex sequence of propagative reactions, lipid hydroperoxide is formed, which then decomposes to the reactive aldehyde malondialdehyde (MDA), and other products indicative of oxidative stress (22).

It is well-known that wildlife translocation is associated with short and long-term stress responses (6). To mitigate stress perception and moderate the potentially adverse effects of these stress responses, rhinoceroses are frequently tranquilized during capture and transport—components of translocation (6, 23). Azaperone, a butyrophenone, is most commonly used in rhinoceroses and functions both as an “opioid-synergist” during capture, and as a short duration tranquilizer during transport (23, 24). Its behavior-dampening effects are mediated primarily by blockade of dopamine receptors in the central nervous system (25). Midazolam is being used more often in rhinoceros translocation as it is believed to have greater anxiolytic effects than azaperone (26, 27). It is a benzodiazepine derivative which modulates the gamma-aminobutyric acid (GABA)_A_ receptor in the central nervous system, producing powerful anxiolytic, amnestic, hypnotic, and sedative effects (28). Benzodiazepines also bind to peripheral benzodiazepine receptors (PBR), or translocator proteins (18 kDa), which are widely expressed throughout the body (29). Interestingly, PBR densities are particularly rich in steroidogenic tissues, specifically in the adrenal gland, and may therefore have a direct modulating effect on the stress response and associated immunological changes (29, 30).

The aim of this study was to investigate stress responses to capture and transport in wild white rhinoceros bulls by quantifying hematological (blood cell count) and immunological (acute phase reactants, lipid peroxidation products) changes as well as specific stress response indicators (adrenal hormone concentrations, N:L ratio, LCC). We investigated whether midazolam was better able to mitigate these responses compared to azaperone. We hypothesized that over time rhinoceroses would experience an increase in adrenal hormones and N:L ratio, a decrease in LCC, an increase in positive and decrease in negative acute phase reactants and an increase in lipid peroxidation products. Because of the greater anxiolytic effects of the midazolam, we hypothesized that rhinoceroses treated with this drug would experience smaller changes in these variables compared to rhinoceroses treated with azaperone.

## Materials and Methods

Twenty three wild white rhinoceroses were road-transported 280 km within the Kruger National Park (24.9948° S, 31.5969° E; altitude 317 m), South Africa, for reasons unrelated to the study. The individuals and number of animals subjected to the translocation were chosen by park authorities based on important population management decisions. Only sub-adult males were translocated allowing for a homogenous study group. Four animals (three on one occasion) were captured and transported at a time, resulting in six translocation events taking place over a 3 week period in July 2018 (Southern hemisphere wintertime). All procedures were performed according to the Standard Operating Procedure for the Capture, Transport and Maintenance in Holding Facilities of Wildlife as approved by the South African National Parks (SANParks) Animal Use and Care Committee (AUCC). International Air Transport Association compliant transport crates were used and practical guidelines for transport of live wild animals (31) and rhinoceroses (32, 33) were followed. The study was approved by the University of Pretoria Animal Ethics and Research Committee (V067-17) and SANParks AUCC (009/17).

### Capture and Transport

#### Capture

Rhinoceroses were darted remotely from a helicopter into the gluteal muscle using 3.0 mL plastic darts with a 60 mm uncollared needle (Dan-Inject®, International S.A., Skukuza 1350, South Africa). Two different immobilization protocols were used alternately: either etorphine (etorphine hydrochloride 9.8 mg/mL, Captivon® Wildlife Pharmaceuticals, Karino, South Africa) combined with azaperone (azaperone tartrate 50 mg/mL, Wildlife Pharmaceuticals) (group A, *n* = 11), or etorphine combined with midazolam (midazolam hydrochloride 50 mg/mL, Dazonil® Wildlife Pharmaceuticals) (group M, *n* = 12). Etorphine doses were based on standardized estimated weight categories: 1,250–1,500 kg = 3 mg; 1,500–1,750 = 3.5 mg; 1,750–2,000 = 4 mg, aiming to administer 2 μg/kg. Azaperone or midazolam were administered at five times the etorphine dose in mg. These doses have been used in clinical practice and deemed to be optimally effective as “opioid-synergists” in white rhinoceroses (24). Once immobilized, rhinoceroses were positioned in lateral recumbency and a blood sample was immediately collected from the cephalic vein (time capture sample = TC). The auricular skin was aseptically prepared and a 16 gauge 20 cm over-the-wire intravenous catheter (Arrow®, PA 19605 USA) inserted into an auricular vein using the Seldinger technique. Heart rate, respiratory rate, and body temperature were monitored throughout the 30 min procedure and oxygen was delivered at a constant rate of 10 L/min by nasal insufflation. Once the catheter was in place, butorphanol (5 mg for every mg of etorphine; butorphanol tartrate 50 mg/mL, Wildlife Pharmaceuticals) was administered intravenously to partially antagonize the μ-opioid receptor effects of the etorphine (34) and allow for loading of the rhinoceros into the transport crate. An intravenous bolus of diprenorphine (3 mg for every mg of etorphine; diprenorphine hydrochloride 12 mg/mL Activon® Wildlife Pharmaceuticals) was administered once the animal was in the crate to further antagonize the immobilizing, but not the sedative, effects of the etorphine (35).

#### Transport

Once all four rhinoceroses (three rhinoceroses on one occasion) had been captured and loaded into the transport crates, a venous blood sample was collected from the auricular catheter at the start of transport (time 0 h transport sample = T0). For practical reasons, we re-administered midazolam at the same dose and time interval as recommended and clinically used for azaperone during rhinoceros transport (23). Specifically, azaperone (group A) or midazolam (group M) was re-administered intramuscularly at 25 times the etorphine dose, in mg, at the start of transport, and 2–4 h later. The destination was reached after 6 h, a final blood sample was collected (time 6 h transport sample = T6) and the auricular catheter removed. Naltrexone (80 mg; naltrexone hydrochloride 50 mg/mL, Trexonil® Wildlife Pharmaceuticals) was administered intravenously to fully antagonize any residual etorphine effects prior to releasing the rhinoceroses back into the wild.

### Blood Sample Collection and Analysis

#### Complete Blood Cell Count

Blood directly collected into ethylenediaminetetraacetic acid (EDTA) tubes (BD Vacutainer; Becton and Dickinson, Plymouth, UK) was stored in a cooler box with ice packs during transport and was analyzed at the release site with the fully-automated Abaxis® VetScan HM5 differential hematology analyzer (Abaxis Global Diagnostics, Griesheim, Germany). One level of commercial quality control material was run each day of sample analysis and results were within the manufacturer's target range. The device measured and, or, calculated: hematocrit (HCT) red blood cell count (RBC), hemoglobin concentration (HGB), mean cell volume (MCV), mean cell hemoglobin (MCH), mean cell hemoglobin concentration (MCHC), red blood cell distribution width (RDW), plateletcrit (PCT), platelet count (PLT), mean platelet volume (MPV), platelet distribution width (PDW), and white blood cell count (WBC). Packed cell volume (PCV) was determined manually. The calculated HCT of the Abaxis was compared to the manual PCV as reference, using different settings, and if there was a discrepancy larger than 5%, the analysis was repeated. The Abaxis “cow” setting was used as it demonstrated the best match between calculated HCT and manual PCV. In order to examine cell morphology, and because automated leukocyte differential counting has not been validated for this species (36, 37), blood smears were made by using the wedge method (38) and examined at a later point by an experienced clinical pathologist (EHH). The relative proportion of each WBC type (differential count) was measured by light microscope examination of 100 leukocytes in a modified Romanowsky stained blood smear; immature neutrophils (BANDS%), mature neutrophils (SEG%), lymphocytes (LYM%), monocytes (MON%), and eosinophils (EOS%) were counted. Absolute neutrophil (NEU), and lymphocyte (LYM) counts were calculated by multiplying the sum of BANDS% plus SEG% (NEU), and LYM% (LYM), from the 100-cell count, with the total Abaxis WBC count. The N:L ratio was calculated by dividing NEU by LYM.

#### Leukocyte Coping Capacity

Immediately after blood collection into lithium-heparinized blood tubes (BD Vacutainer), LCC measurements were carried out in the field following the protocol published in Huber et al. (16, 39). Briefly, by adding luminol, the chemiluminescence of phorbol 12-myristate 13-acetate (PMA) stimulated and unstimulated samples (control) was measured using a portable chemiluminometer (Junior LB 9509, E G & G Berthold, Germany). The area under the response curve was calculated from PMA-stimulated samples and corrected for the PMA-unstimulated measurements. We then corrected the area under the curve by the absolute neutrophil count at each time sample point to examine the effect of ROS production per neutrophil and to control for a potential mass effect.

#### Epinephrine

Immediately after collection, EDTA blood tubes were centrifuged in a centrifuge cooled to 4°C. Plasma was pipetted into cryovials and immediately snap-frozen in liquid nitrogen. Samples were subsequently stored at −80°C for 2 weeks and shipped to the Analytical Technical Laboratory of the Faculty of Health Sciences, North-West University, South Africa, using dry-ice. Epinephrine concentrations were determined using the sample preparation technique as described by de Villiers et al. (40) and analyzed with a chromatographic system consisting of an Ultimate 3000 UHPLC system, equipped with an ISO-3100SD isocratic pump and WPS-3000TSL analytical autosampler, coupled to an ECD-3000RS rapid separation electrochemical detector with 2-Channel Coulometric Cell 6011RS and Chromeleon® chromatography management system version 7.2 (all obtained from Thermo Fisher Scientific, Waltham, MA USA). The limit of detection was 5 nmol/l plasma epinephrine. Values below this limit were included as “zero” for the analysis of results.

#### Lipid Peroxidation Products

Duplicate snap-frozen EDTA plasma samples were shipped on dry-ice to the Chemical Pathology Laboratory of the Faculty of Health Sciences, University of Cape Town. Plasma triglyceride and phospholipid concentrations were determined using enzymatic colorimetric kits (WAKO Chemicals GmbH, Neuss, Germany) in a SPECTRA-maxPLUS-384 spectrophotometer (Molecular Devices Corporation, Labotec Industrial Technologies, South Africa). Concentrations of conjugated dienes (CD) and thiobarbituric acid reactive substances (TBARS) were analyzed using spectrophotometric methods and measured in the spectrophotometer as above. Conjugated dienes were measured at 234 nm after appropriate dilution in cyclohexane (Spectrosol) as described by Pryor and Castle (41) and Esterbauer et al. (42). Thiobarbituric acid reactive substances were measured at 532 nm after being prepared as described by Nduhirabandi et al. (43). Conjugated dienes and TBARS measurements were corrected per total lipid concentration (the sum of triglycerides and phospholipids) to examine the effect of the fatty acids on reactive oxygen species production.

The antioxidant capacity of the plasma was assessed by the oxygen radical absorbance capacity (ORAC) method described by Cao et al. (44) and Huang et al. (45). Fluorescence was measured using the Varian Cary Eclipse fluorescence spectrophotometer (Varian Australia Pty Ltd) at an excitation wavelength of 485 nm and emission wavelength of 520 nm.

#### Acute Phase Reactants and Cortisol

Blood directly collected into sodium-citrate (CTAD) and serum tubes (BD Vacutainer) was stored in a cooler box with ice packs during transport and centrifuged at the release site. Serum and plasma were aliquoted and stored at −80°C until analysis at the clinical pathology laboratory of the Onderstepoort Veterinary Academic Hospital, University of Pretoria. Fibrinogen was determined from the CTAD plasma with the modified Clauss method on an ACL Elite automated coagulometric analyzer (Instrumentation Laboratory, Bedford, MA, USA). Serum haptoglobin was determined by the hemoglobin-binding method using a commercial kit (PHASE Haptoglobin Assay, Tridelta Development Limited, Kildare, Ireland) on a Cobas Integra 400 Plus automated biochemistry analyzer (Roche Diagnostics Ltd., Rotkreuz, Switzerland) (46). Serum albumin and iron concentrations were measured using commercially available kits on the Cobas Integra 400 Plus. Serum cortisol concentrations were assessed by a chemiluminescent immunoassay using the Immulite/Immulite 1000 Cortisol® following manufacturer's instructions (Siemens Healthcare, Erlangen, Germany). All analyzers were maintained and kits were calibrated according to manufacturer's instructions; two levels of commercial quality control material were analyzed before each assay run and results were within the laboratory's predetermined target ranges.

### Data Analysis

Variables were divided into three multivariate datasets for the interpretation of results: [1] hematological response (RBC, HGB, PCV, MCV, MCH, MCHC, RDW, PCT, PLT, MPV, PDW, WBC, BANDS%, SEG%, LYM%, MON%, EOS%, NEU, LYM), [2] stress response (epinephrine, cortisol, N:L ratio, LCC), [3] immunological response (fibrinogen, haptoglobin, albumin, iron, triglycerides, phospholipids, CD, TBARS, ORAC).

Statistical analysis was performed with the software R version 3.6.1 (47). Data were assessed for normality by calculating descriptive statistics and plotting of histograms. Mean ± standard deviations (SD) were calculated for each variable per sample time point and group and interval plots were generated for descriptive purposes. A general linear mixed model (fixed factors: sample time point and group; random factors: individual rhinoceros; interactions: sample time point x group) was used to compare changes over time and between groups. Start of transport (T0) and group A were used as reference category to [1] better differentiate the effects of capture (TC–T0) from the effects of transport (T0–T6) and because [2] azaperone is the drug that is currently most commonly added to the etorphine for rhinoceros capture and transport. Pearson's correlations were performed to investigate correlations between stress response indicators. The Bonferroni correction for multiple correlations was applied. Differences were considered significant when *p* ≤ 0.050.

## Results

All rhinoceroses survived capture and transport without any signs of injuries or capture related pathologies. Rhinoceroses were weighed when placed in the transport crate and the drug doses used for the immobilization and sedation recalculated on a per kilogram basis. The animals' weight ranged from 1,155 to 2,046 (1,547 ± 238) kg, which was slightly less than estimated. In group A, etorphine and azaperone were administered at 2.49 ± 0.38 and 12.27 ± 2.09 μg/kg, respectively. In group M, etorphine and midazolam were administered at 2.58 ± 0.37 and 12.07 ± 1.86 μg/kg, respectively. During transport azaperone was administered at 62.38 ± 9.54 μg/kg, and midazolam at 64.61 ± 9.28 μg/kg. As we could only capture one rhinoceros at a time, the individuals captured at first had to wait in the transport crates until all four animals had been caught and transport was started. This limitation resulted in a time-lag from TC to T0 between individuals, which was 189 ± 84 min in group A and 117 ± 73 min in group M (*p* = 0.031). Descriptive analysis of results presenting mean ± standard deviation of the measured variables (PCV, RBC, HGB, PCV, MCV, MCH, MCHC, RDW, PCT, PLT, MPV, PDW, WBC, BANDS%, SEG%, LYM%, MON%, EOS%, NEU, LYM, epinephrine, cortisol, N:L ratio, LCC, triglycerides, phospholipids, CD, TBARS, ORAC, fibrinogen, haptoglobin, albumin, iron) at TC, T0 and T6 is shown in Supplementary Table 1 for the different groups. Model-based analysis presenting coefficient estimates and corresponding standard errors and *p*-values is provided in Supplementary Table 2.

### Hematological Response

**Figure 1:**
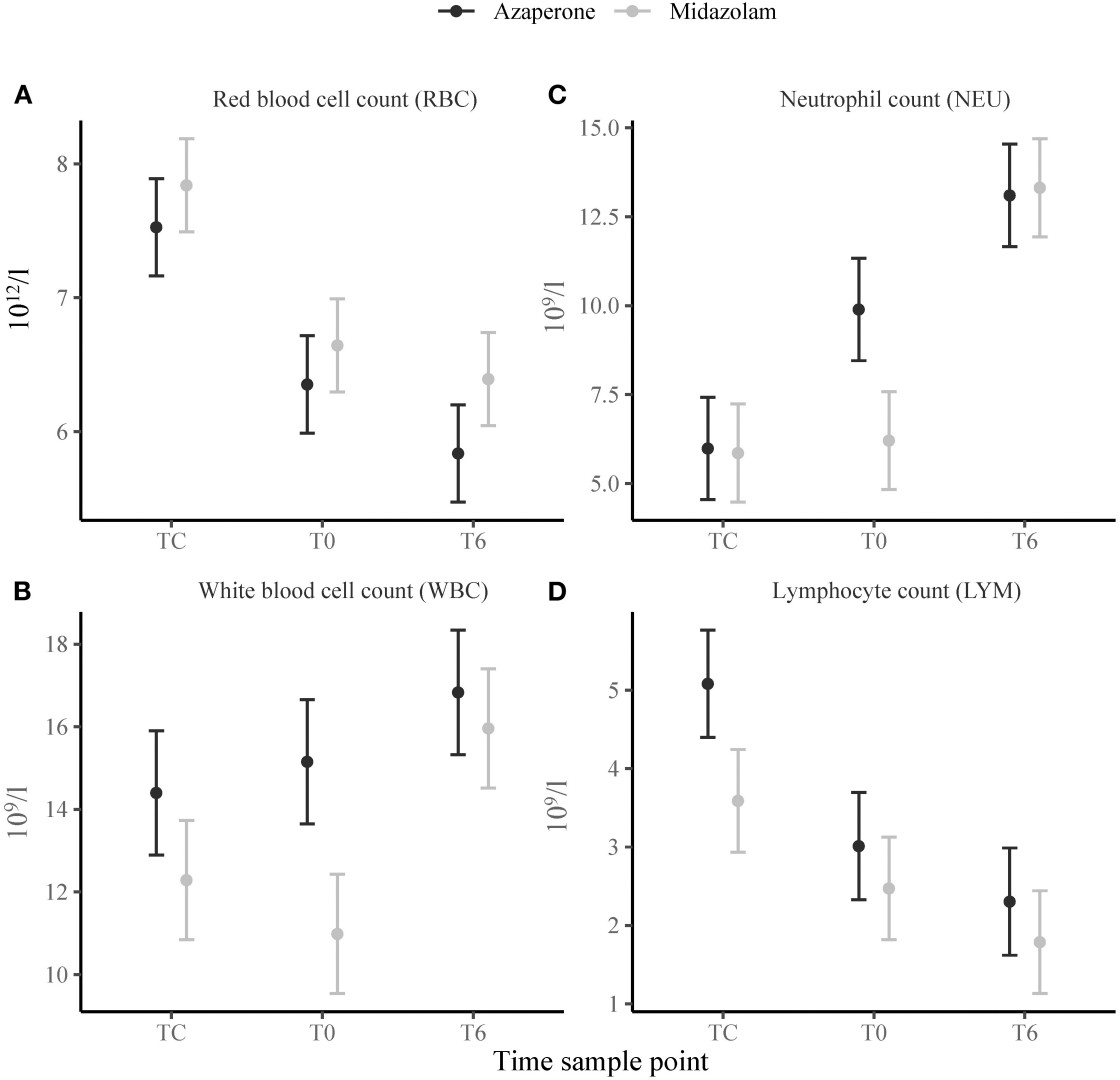
Means ± standard error of selected hematological variables: **(A)** Red blood cell count (RBC), **(B)** white blood cell count (WBC), **(C)** neutrophil count (NEU), **(D)** lymphocyte count (LYM) in white rhinoceroses captured and transported with either azaperone or midazolam. Time sample points: capture (TC), start of transport (T0), and 6 (T6) h of transport.

We found a strong and significant main effect of time in the erythron. Packed cell volume, RBC (Figure 1A) and HGB decreased from TC to T0 (*p* < 0.001) and from T0 to T6 (*p* = 0.002, *p* < 0.001 and *p* = 0.002, respectively). The red cell indices MCH, MCHC and RDW decreased (*p* < 0.001), and MCV increased (*p* = 0.004), from TC to T0, but did not change from T0 to T6. There were no significant effects of midazolam or midazolam x time (Supplementary Table 2). We also found no significant main effects of midazolam, or time, in the thrombon. However, there was a positive interaction effect of midazolam and T6 for PCT and PLT (*p* = 0.030 and *p* = 0.036, respectively).

The leukon showed a strong and significant main effect of time. White blood cell count increased from T0 to T6 (*p* = 0.018) (Figure 1B). There were more BANDS% at TC than at T0 (*p* = 0.034). Neutrophils increased from TC to T0 (SEG% and NEU *p* < 0.001) and T0 to T6 (SEG% *p* = 0.001 and NEU *p* < 0.001) (Figure 1C). Lymphocytes decreased from TC to T0 (LYM% and LYM *p* < 0.001) and T0 to T6 (LYM% *p* = 0.019) (Figure 1D). Similarly, EOS% decreased from TC to T0 (*p* = 0.001) and T0 to T6 (*p* = 0.003). White blood cell count and NEU revealed a negative (*p* < 0.001) main effect of midazolam with the WBC and NEU being lower in group M than A. EOS% showed a positive main effect of midazolam (*p* = 0.034). There were a number of significant interaction effects between group and time. For WBC, SEG% and NEU, there was a positive interaction effect of midazolam and TC (*p* = 0.033, *p* = 0.010 and *p* = 0.002) and midazolam and T6 (*p* = 0.001, *p* = 0.010 and *p* < 0.001). We found a negative interaction effect of midazolam and TC for LYM and MON% (*p* = 0.033 and *p* = 0.041) and of midazolam and T6 for MON% and EOS% (*p* = 0.043 and *p* = 0.019).

### Stress Response

**Figure 2:**
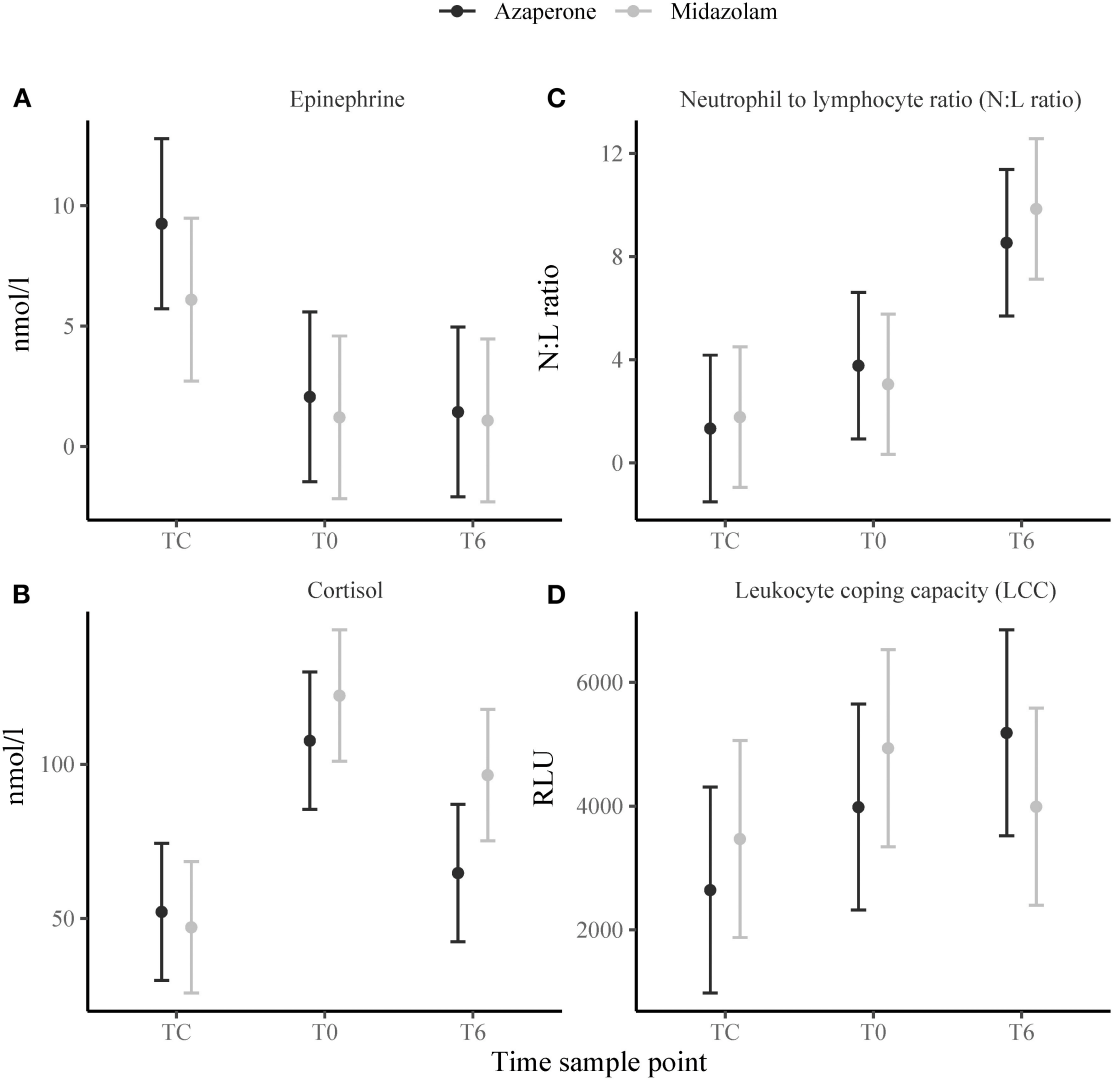
Means ± standard error of stress response indicators: **(A)** epinephrine, **(B)** cortisol, **(C)** neutrophil to lymphocyte ratio (N:L ratio), **(D)** leukocyte coping capacity (LCC) in white rhinoceroses captured and transported with either azaperone or midazolam. Time sample points: capture (TC), start of transport (T0), and 6 (T6) h of transport.

Time sample point had significant effects on plasma epinephrine concentrations, cortisol and N:L ratio. At TC, plasma epinephrine concentrations were above the detection limit of 5 nmol/l in 12 rhinoceroses (group A, *n* = 7; group M, *n* = 5) (Figure 2A), but decreased to below this detection limit by T0 (*p* < 0.001). Only in 4 of these 12 animals (two from each group) plasma epinephrine concentrations could still be detected at T0 and T6. Serum cortisol concentrations increased from TC to T0 (*p* < 0.001) and decreased between T0 and T6 (*p* = 0.002) (Figure 2B). The N:L ratio increased from T0 to T6 (*p* = 0.013) (Figure 2C). Time had no significant effect on LCC (Figure 2D). There were no significant main effects of midazolam or interaction effects of midazolam and time on any of these variables. Results of the Pearson correlation showed that there were also no significant correlations between N:L ratio, LCC, epinephrine and cortisol concentrations (Table 1).

**Table 1:** Association among neutrophil-to-lymphocyte ratio (N:L ratio), leukocyte coping capacity (LCC), plasma epinephrine, and cortisol measurements in 23 white rhinoceros bulls during capture and transport.

	**N:L ratio**	**LCC**	**Epinephrine**	**Cortisol**
N:L ratio		*p* = 1.000	*p* = 0.152	*p* = 1.000
LCC	*r* = −0.069		*p* = 0.658	*p* = 1.000
Epinephrine	*r* = −0.269	*r* = −0.194		*p* = 0.130
Cortisol	*r* = −0.010	*r* = 0.125	*r* = −0.276	

*Pearson correlation coefficients (r) and p values are shown*.

### Immunological Response

**Figure 3:**
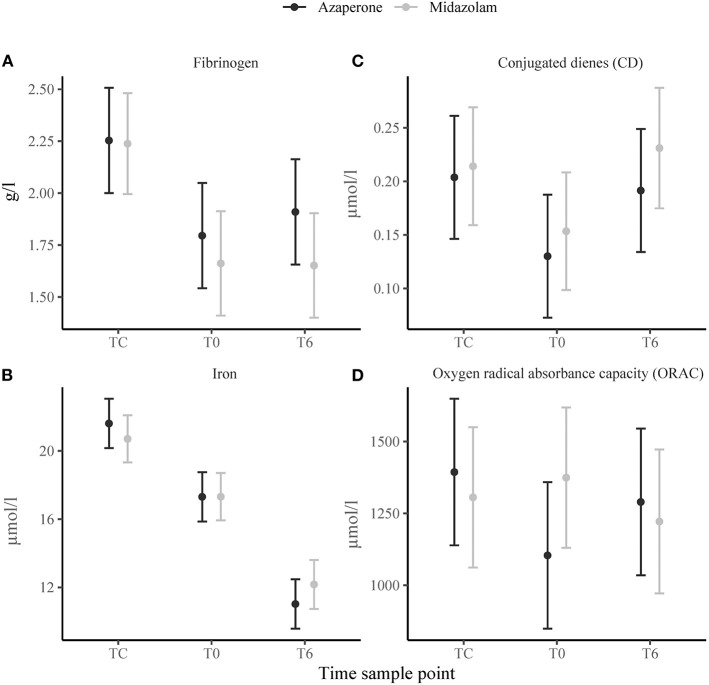
Means ± standard error of immunological variables: **(A)** fibrinogen, **(B)** iron, **(C)** conjugated dienes (CD), **(D)** oxygen radical absorbance capacity (ORAC) in white rhinoceroses captured and transported with either azaperone or midazolam. Time sample points: capture (TC), start of transport (T0), and 6 (T6) h of transport.

There was a significant effect of time on serum fibrinogen (Figure 3A), haptoglobin and albumin concentrations, which were higher at TC compared to T0 (*p* = 0.002, *p* = 0.022, and *p* < 0.001, respectively) and did not change thereafter. Serum iron concentrations gradually decreased from TC to T0 (*p* < 0.001) and T0 to T6 (*p* < 0.001) (Figure 3B). Plasma triglyceride and phospholipid concentrations increased from TC to T0 (*p* = 0.021 and *p* = 0.028, respectively), but triglyceride concentrations decreased from T0 to T6 (*p* < 0.001). Conjugated dienes decreased from TC to T0 (*p* = 0.004), but increased from T0 to T6 (*p* = 0.014) (Figure 3C). Time had no significant effect on TBARS concentrations, but TC had a positive effect on ORAC (*p* = 0.011). The trend of ORAC over time seemed to differ between the two rhinoceros groups (Figure 3D) with group A experiencing a decrease from TC to T0. There were no significant main effects of group on any of these variables. However, we found a negative significant interaction effect of midazolam and TC (*p* = 0.023) and midazolam and T6 (p = 0.034) for ORAC and a positive interaction effect of midazolam and T6 (*p* = 0.043) for albumin.

## Discussion

Rhinoceroses mounted a stress response to capture and transport with highest plasma epinephrine concentrations at TC, peak serum cortisol concentrations at T0, and an increase in N:L ratio from T0 to T6. A gradual decrease in serum iron concentrations over time indicated that rhinoceroses likely mounted an acute phase reaction. Increased lipid peroxidation products at TC and T6, compared to T0, suggested rhinoceroses experienced oxidative stress at capture and with increasing transport duration. Midazolam had no effect on the magnitude of the adrenal stress response, but appeared to directly influence leukocyte numbers. The interaction of midazolam and time had a strong effect on leukocyte numbers and some lipid peroxidation products suggesting an immunomodulating effect of the benzodiazepine.

### Hematological Response

Rhinoceroses immobilized with etorphine based drug-combinations are known to exhibit severe tachycardia and systemic hypertension resulting from the effects of the potent opioid combined with sympathetic activation (48, 49). Elevated plasma epinephrine concentrations at TC compared to the other sample time points indicated that our rhinoceroses likely experienced tachycardia and hypertension, which for logistical reasons, we were not able to measure. This tachycardia and hypertension could have caused an increase in hydrostatic pressure and movement of fluid from the vessels into the extravascular space (50, 51) resulting in the higher PCV, RBC, and HGB at TC compared to the other sample time points. This process is referred to as stress-hemoconcentration and has been linked to acute psychological stress in humans (50, 52). However, splenic contraction in response to the circulating catecholamine concentrations cannot be excluded and likely also played a role in our rhinoceroses. Similar to horses, a species related to the rhinoceros, the rhinoceros's spleen could represent a reservoir of reds blood cells, which rapidly enter the circulation during a fight or flight response in order to enhance oxygen transport capacity (51, 53). These red blood cells would be older and smaller in size than normal circulating red blood cells (54), which could explain the higher RDW and MCHC, and lower MCV at TC compared to the other sample time points. Additionally, splenic release of immature neutrophils may have caused the concurrent elevation of this white blood cell-type at TC (55).

Plasma cortisol concentrations increased from TC to T0 and likely caused the observed sustained decrease in lymphocytes and eosinophils, and increase in mature neutrophils over time. In response to glucocorticoids, circulating lymphocytes and eosinophils adhere to the vascular endothelium and transmigrate from the circulation into other tissues, such as lymph nodes, spleen, bone marrow and skin, where they are sequestered (14). Neutrophils in contrast, migrate from the bone marrow into the blood and shift from the marginating to circulating blood pool (11, 14). Rhinoceroses from group M had, at all times, lower WBC than rhinoceroses from group A. In human leukocytes, peripheral benzodiazepine binding receptors (PBRs) have been identified on the plasma membrane and are suggested to play a role in neuroendocrine-immunomodulation (30). Monocytes and lymphocytes in particular appear to express an abundance of these receptors (56). During an initial stress response, circulating monocytes and lymphocytes produce proinflammatory cytokines, which assist in attracting neutrophils (57, 58). In midazolam-sedated rhinoceroses, the increase in neutrophil concentrations in the circulating pool appeared to be delayed compared to azaperone-sedated rhinoceroses (Figure 2C). This delay could have resulted from a monocyte and lymphocyte inhibitory effect of the midazolam reducing the cells' capacity to attract neutrophils and should be investigated in future studies (56). Lower monocyte and lymphocyte concentrations at TC in the rhinoceroses of this group could have contributed to this effect as well as the time lag from TC to T0 between group A and M. Due to practical reasons, because we could not capture more than one rhinoceros at a time, it was not possible to standardize this time lag for all animals, which represents an unavoidable study limitation.

### Stress Response

Similar to reports in other transported wild mammals (59–61), the N:L ratio gradually increased in the studied rhinoceroses. The magnitude of increase in N:L ratio is believed to be proportional to the magnitude of glucocorticoid release (14). However, we found no correlation between N:L ratio and the other stress response variables. Esteruelas et al. (62) report a similar outcome on the same stress parameters in Scandinavian brown bears captured via darting from a helicopter. The reason for this discrepancy is that the change in N:L ratio occurs over a different time scale than the change in adrenal hormone concentrations (63). Epinephrine concentrations were highest at TC suggesting a rapid and short duration release of catecholamines within the acute period of the stress response (64). Cortisol concentrations in contrast, increased from TC to T0 and decreased between T0 and T6. A similar trend has been described in other white rhinoceroses with plasma cortisol concentrations increasing for 100 min after adrenocorticotropic hormone injection (65). In horses, a species related to the rhinoceros, elevations in N:L ratio were found to occur only 4 h after cortisol injection (66). In our animals, the N:L ratio increased from T0 to T6, suggesting a similar timescale. Despite the differences in leukocyte differential counts between the two rhinoceros groups, the N:L ratio did not differ. This finding agrees with the fact that plasma epinephrine and serum cortisol concentrations also did not differ between the two groups indicating that the administered dose of midazolam did not reduce HPA activation compared to azaperone.

The second and less frequently applied immunological indicator of a stress response used in our study was LCC. Leukocyte coping capacity provides a more integrated measure of a stress response by quantifying some of the complexity of action and reaction of neutrophils to a multitude of stress signals (67). In our rhinoceroses, time had no significant effect on this variable. Using a similar assay protocol, Kruger et al. (68) also found no significant differences in LCC in white rhinoceroses between capture (anesthetic induction) and immediately after loading into a transport crate (20–30 min later) (68). These results do not necessarily mean that capture and transport are not stressful or had no effect on the potential of circulating neutrophils to produce ROS in our rhinoceroses. McLaren et al. (15) measured lower LCC in transported compared to non-transported wild badgers (*Meles meles*) indicating greater stress levels in the transported group (15, 60). Taking the other stress response variables and their temporal dynamics into account (64), it is likely that in our rhinoceroses LCC was decreased at TC and, due to the length of the stress response, did not fully recover during transport. Comparison of LCC measurements with a non-captured, or at least captured but non-transported control group, would have been indicated to disentangle the effect of capture and transport on neutrophil function.

Whilst this was, for animal welfare reasons, not possible, we compared LCC in transported rhinoceroses supplemented with azaperone to midazolam. It has been shown that some anesthetic agents have the potential to directly decrease neutrophil oxidative burst capacity (69). In horses, midazolam induced a dose-dependent reduction on peripheral blood neutrophil function (70). As with the other stress response variables, midazolam had no significant effect on LCC indicating that, compared to azaperone, the administered dose did not meaningfully alter neutrophil function. Future studies are required to investigate dose and time-dependent effects of the administered sedatives on neutrophil function in rhinoceroses and their clinical importance during translocation.

### Immunological Response

In the white rhinoceros, fibrinogen and haptoglobin are positive APRs, which increase, and albumin and iron are negative APRs which decrease during an acute phase reaction (46). In the rhinoceroses of this study, fibrinogen and haptoglobin concentrations did not increase; instead, they decreased from TC to T0, as did albumin. These decreases likely represented relative plasma concentration changes caused by fluid shifts associated with the stress hemoconcentration at TC (50). During stress hemoconcentration, molecules larger than 69 kDa, such as most acute phase proteins, are unable to passively follow the plasma through the capillary pores and therefore increase in concentration (50). Serum iron is a small molecule and therefore not affected by stress hemoconcentration. It has been shown to be a reliable indicator for inflammation in horses and demonstrated excellent diagnostic accuracy in rhinoceroses (46). We therefore believe that the observed gradual decrease in serum iron concentrations, despite the lack of increase in the measured positive APRs, indicates that animals mounted an acute phase reaction. Further research is required to fully elucidate APRs in white rhinoceroses and their role during an acute stress response when fluid shifts are expected.

An acute phase reaction is often accompanied by alterations in plasma oxidants and antioxidants involving lipid peroxidation and oxidative stress (12). Lipid peroxidation can be identified at different stages by measuring: (1) oxidation of unsaturated fatty acids (e.g., in triglycerides and phospholipids), (2) increases of primary peroxidation products (e.g. CD), (3) increases of secondary peroxidation products [e.g., MDA (TBARS)] (20), or (4) the consumption of plasma antioxidant substances (e.g., ORAC) (20). Decreased unsaturated fatty acid concentrations and increased CD concentrations at TC and T6 compared to T0 indicated that lipid peroxidation likely occurred at these time points. Plasma MDA concentrations, measured as TBARS, did not change over time, but because MDA is quickly metabolized it is not an ideal biomarker for oxidative stress (20). Interestingly, the trend of the oxygen radical absorbance capacity (ORAC) differed between the two rhinoceros groups. Azaperone-sedated rhinoceros experienced a decrease in ORAC from TC to T0 indicating that radical-scavenging antioxidants were consumed in response to ROS generation (20). In midazolam-sedated rhinoceros ORAC did not drop at this time point, perhaps because of the delayed increase in ROS producing neutrophils. Although midazolam had no significant effect on ORAC, the interaction of midazolam and time had a negative effect. This finding was surprising, because benzodiazepines have been found to inhibit mitochondrial ROS production in endothelial and neural cells *in vitro* (71), and should therefore protect from oxidative stress, in contrast to butyrophenones, which have been linked to increases in ROS production and cytotoxicity (72). However, the clinical relevance of these effects in the rhinoceros is not yet understood and requires further investigation. Oxidative stress as well as translocation are known to increase the susceptibility to pathogens in wild and farm animals (6, 73). In women, oxidative stress has been directly linked to chronic stress exposure (74) and wildlife studies are increasingly using oxidative stress as a cumulative indicator of animal welfare (75). Whether the rhinoceroses in this study started experiencing chronic stress is unclear and remains the subject of future studies. New, non-invasive markers of oxidative stress could be implemented in post-release monitoring and investigated together with spatial, behavioral, hormonal, and disease measurements to identify animals with an increased risk of developing morbidity after translocation.

## Conclusions

Rhinoceroses in this study mounted an adrenal stress response to capture and transport and experienced hemoconcentration, an increase in N:L ratio, an acute phase reaction and oxidative stress. It is important to understand that these immunological changes have a protective purpose in an acute situation and prepare the immune system for challenges that may be imposed by a stressor (9, 14). In chronic situations, which persist from days to months, these adaptive immunological responses may become harmful and increase the susceptibility to disease (6, 9). In our rhinoceroses, midazolam, compared to azaperone, appeared to influence the white cell response, but not the stress response *per se*. Several studies have identified an increased risk of developing disease (e.g., pneumonia, orthopox virus infection) in patients exposed to benzodiazepines (76–78). This risk, together with the fact that wildlife translocation has already been linked to chronic stress and morbidity (5, 6, 79), could cause concerns about the repetitive use of midazolam for rhinoceros translocation. Therefore, further research that includes extensive post-release monitoring should be done to investigate if rhinoceroses develop an increased risk for disease after translocation when midazolam is used. Dose- and time-dependent immunomodulating effects of this drug need to be explored, as well as a potential anti-inflammatory effect. Midazolam improves lactic acidosis during rhinoceros immobilization (80) and may reverse behavioral deficits associated with chronic stress (81). These benefits may be clinically more important than potential immunological side effects and need to be explored. The ultimate goal of this study was to help improve the outcome of rhinoceros translocation and contribute toward conservation of the species, by better controlling the stress response, using midazolam instead of azaperone. Although midazolam was not able to do so, the information gained from this research has paved the way for further studies investigating the interface between the stress response, the immune system and the development of disease after capture and transport in wild white rhinoceroses.

## Data Availability Statement

The raw data supporting the conclusions of this article will be made available by the authors, without undue reservation, to any qualified researcher.

## Ethics Statement

The animal study was reviewed and approved by University of Pretoria Animal Ethics and Research Committee (V067-17) and the South African National Parks Animal Use and Care Committee (009/17).

## Author Contributions

FP, EH, and LM designed the experiment together with PB. PB took veterinary care of the rhinoceros. FP, PB, EH, NH, and LM collected the data. EH conducted and coordinated blood sample analysis (hematology, clinical chemistry analytes). NH conducted LCC measurements. FV conducted epinephrine measurements. DB performed the lipid peroxidation assays and analysis of oxidative stress biomarkers. Data analysis and preparation was done by FP. FP wrote the manuscript together with all co-authors. All authors approved the final manuscript.

## Conflict of Interest

The authors declare that the research was conducted in the absence of any commercial or financial relationships that could be construed as a potential conflict of interest.
