# Supplementary material for: A Comparison of Hematological, Immunological, and Stress Responses to Capture and Transport in Wild White Rhinoceros Bulls (Ceratotherium simum simum) Supplemented With Azaperone or Midazolam
Source: Front Vet Sci. 2020 Oct 20;7:569576. doi: 10.3389/fvets.2020.569576 (PMC7606872; doi:10.3389/fvets.2020.569576)
Supplement: Supplementary file 1 [file Data_Sheet_1.pdf]

Supplementary Material

1 Supplementary Tables

Table S1. Mean ± standard deviation for measured variables in rhinoceros captured and transported with either azaperone (group A) or midazolam (group M). Time sample points: capture (TC), start of transport (T0), and six hours of transport (T6).

Variable	Unit	Group	Time sample point		
			TC	T0	T6
**Hematological response**					
PCV	%	A	46 ± 3	37 ± 5	34 ± 3
		M	45 ± 3	37 ± 4	36 ± 2
RBC	10^{12}/l	A	7.53 ± 0.36	6.35 ± 0.46	5.84 ± 0.56
		M	7.84 ± 0.52	6.64 ± 0.97	6.39 ± 0.52
HGB	g/l	A	155 ± 8	127 ±8	117 ±9
		M	158 ± 8	131 ± 17	127 ± 9
MCV	fl	A	69 ± 5	70 ± 4	70 ± 4
		M	68 ± 5	69 ± 4	69 ± 5
MCH	pg	A	20.6 ± 1.5	20.1 ± 1.6	20.1 ± 1.4
		M	20.2 ± 1.3	19.8 ± 1.1	19.9 ± 1.2
MCHC	g/l	A	301 ± 7	287 ± 9	288 ±11
		M	300 ± 10	289 ± 11	288 ± 7
RDW	%	A	22.2 ± 1.1	21.4 ± 1.1	21.5 ± 1.0
		M	22.4 ± 0.8	21.6 ± 0.7	21.5 ± 0.6
PCT	%	A	0.09 ± 0.04	0.08 ± 0.04	0.07 ± 0.04
		M	0.08 ± 0.02	0.07 ± 0.03	0.09 ± 0.04
PLT	10^9/l	A	149 ± 59	128 ± 59	108 ± 64
		M	132 ± 33	103 ± 47	127 ± 33
MPV	fl	A	6.4 ± 0.6	6.2 ± 0.4	6.4 ± 0.3
		M	6.2 ± 0.3	6.3 ± 0.3	6.3 ± 0.3
PDW	%	A	27.6 ± 1.8	26.9 ± 1.3	27.0 ± 1.1
		M	26.9 ± 1.4	27.2 ± 1.5	27.2 ± 1.3
WBC	10^9/l	A	14.40 ± 2.61	15.15 ± 3.20	16.83 ± 2.18
		M	12.29 ± 1.79	10.98 ± 3.09	15.96 ± 1.80
BANDS%	%	A	5 ± 4	3 ± 2	2 ± 2
		M	3 ± 3	2 ± 2	2 ± 1
SEG%	%	A	37 ± 8	61 ± 12	75 ± 14
		M	44 ± 10	53 ± 13	81 ± 6
LYM%	%	A	35 ± 9	20 ± 7	14 ± 7
		M	30 ± 7	24 ± 9	11 ± 5
MON%	%	A	10 ± 3	8 ± 3	7 ± 4
		M	8 ± 3	10 ± 3	5 ± 3
EOS%	%	A	13 ± 4	7 ± 6	2 ± 4
		M	15 ± 5	11 ± 6	0 ± 0
NEU	10^9/l	A	5.98 ± 1.00	9.89 ± 3.39	13.10 ± 3.18
		M	5.85 ± 1.60	6.21 ± 2.53	13.31 ± 1.79
LYM	10^9/l	A	5.08 ± 1.81	3.01 ± 0.97	2.30 ± 1.18
		M	3.59 ± 0.86	2.47 ± 0.92	1.79 ± 0.84

Variable	Unit	Group	Time sample point		
			TC	T0	T6
**Stress response**					
Epinephrine	nmol/l	A	9.28 ± 9.77	2.14 ± 4.73	1.51 ± 3.16
		M	6.15 ± 7.99	1.29 ± 2.84	1.16 ± 2.50
Cortisol	mmol/l	A	52.2 ± 21.1	107.6 ± 52.7	64.8 ± 35.6
		M	47.2 ± 16.8	122.2 ± 30.3	96.5 ± 50.2
N:L ratio		A	1.33 ± 0.59	3.77 ± 2.25	8.54 ± 8.14
		M	1.78 ± 0.84	3.05 ± 2.28	9.85 ± 7.34
LCC	RLU	A	2643 ± 310	3984 ± 1774	5184 ± 3338
		M	3469 ± 2215	4937 ± 4187	3990 ± 2612
**Immunological Response**					
Fibrinogen	g/l	A	2.25 ± 0.34	1.80 ± 0.47	1.91 ± 0.33
		M	2.24 ± 0.26	1.65 ± 0.55	1.64 ± 0.51
Haptoglobin	g/l	A	0.68 ± 1.38	0.47 ± 0.94	0.51 ± 0.96
		M	1.15 ± 1.24	0.84 ± 0.96	0.85 ± 0.95
Albumin	g/l	A	26.6 ± 1.5	24.6 ± 1.0	25.0 ± 0.7
		M	27.4 ± 1.4	24.6 ± 1.8	26.1 ± 1.1
Iron	µmol/l	A	21.6 ± 1.3	17.3 ± 3.1	11.0 ± 2.6
		M	20.7 ± 1.9	17.3 ± 2.3	12.1 ± 2.8
Triglycerides	mmol/l	A	0.36 ± 0.09	0.46 ± 0.13	0.27 ± 0.10
		M	0.37 ± 0.08	0.47 ± 0.16	0.34 ± 0.14
Phospholipids	mmol/l	A	0.11 ± 0.05	0.16 ± 0.07	0.16 ± 0.05
		M	0.11 ± 0.04	0.13 ± 0.06	0.13 ± 0.05
CD	µmol/l	A	0.20 ± 0.10	0.13 ± 0.04	0.19 ± 0.08
		M	0.21 ± 0.11	0.15 ± 0.09	0.21 ± 0.11
TBARS	µmol/l	A	0.00 ± 0.00	0.00 ± 0.00	0.31 ± 1.03
		M	0.51 ± 1.19	0.42 ± 1.44	0.22 ± 0.72
ORAC	µmol/l	A	1394 ± 319	1104 ± 432	1289 ± 644
		M	1305 ± 275	1374 ± 348	1228 ± 445

Abbreviations: packed cell volume (PCV), red blood cell count (RBC), hemoglobin (HGB), mean cell volume (MCV), mean cell hemoglobin (MCH), mean cell hemoglobin concentration (MCHC), red blood cell distribution width (RDW), plateletcrit (PCT), platelet blood count (PLT), mean platelet volume (MPV), platelet distribution width (PDW), white blood cell count (WBC), percentage immature neutrophils (BANDS%), percentage mature neutrophils (SEG%), percentage lymphocytes (LYM%), percentage monocytes (MON%), percentage eosinophils (EOS%), absolute neutrophil count (NEU), absolute lymphocyte count (LYM), neutrophil to lymphocyte ratio (N:L ratio), corrected leukocyte coping capacity (LCC), conjugated dienes (CD), thiobarbituric acid reactive substances (TBARS), oxygen radical absorbance capacity (ORAC).

Table S2. Coefficient estimates (standard errors) and *p*-values for time and group on packed cell volume (PCV), red blood cell count (RBC), hemoglobin (HGB), mean cell volume (MCV), mean cell hemoglobin (MCH), mean cell hemoglobin concentration (MCHC), red blood cell distribution width (RDW), plateletcrit (PCT), platelet blood count (PLT), mean platelet volume (MPV), platelet distribution width (PDW), white blood cell count (WBC), percentage immature neutrophils (BANDS%), percentage mature neutrophils (SEG%), percentage lymphocytes (LYM%), percentage monocytes (MON%), percentage eosinophils (EOS%), absolute neutrophil count (NEU), absolute lymphocyte count (LYM), epinephrine (EPI), cortisol, neutrophil to lymphocyte ratio (N:L ratio), corrected leukocyte coping capacity (LCC), fibrinogen (FGN), haptoglobin (HP), albumin (ALB), iron, triglycerides (TG), phospholipids (PL), conjugated dienes (CD), thiobarbituric acid reactive substances (TBARS), oxygen radical absorbance capacity (ORAC). Time: capture (TC), start of transport (T0), and six (T6) hours of transport. Groups: midazolam (group M), azaperone (group A). Reference category: group A time T0. The star indicates statistical significance ($p < 0.05$).

**Variable (unit)**	**Group M**	**Time TC**	**Time T6**	**Group M :Time TC**	**Group M :Time T6**	**Constant**	**Log Likelihood**	**AIC**
PCV<br>(%)	-0.595<br>(1.369)<br>$p = 0.665$	8.955*<br>(1.128)<br>$p < 0.001$	−3.636*<br>(1.128)<br>$p = 0.002$	−0.246<br>(1.561)<br>$p = 0.875$	2.886<br>(1.561)<br>$p = 0.065$	37.136*<br>(0.989)<br>$p < 0.001$	-168.078	352.155
RBC<br>(10^{12}/l)	0.292<br>(0.252)<br>$p = 0.247$	1.175*<br>(0.140)<br>$p < 0.001$	−0.515*<br>(0.140)<br>$p < 0.001$	0.021<br>(0.194)<br>$p = 0.913$	0.264<br>(0.194)<br>$p = 0.174$	6.352*<br>(0.182)<br>$p < 0.001$	-48.554	113.107
HGB<br>(g/l)	3.386<br>(4.303)<br>$p = 0.432$	27.818*<br>(3.267)<br>$p < 0.001$	-10.455*<br>(3.267)<br>$p = 0.002$	-0.485<br>(4.522)<br>$p = 0.915$	6.205<br>(4.522)<br>$p = 0.171$	127.364*<br>(3.108)<br>$p < 0.001$	-237.942	491.883
MCV<br>(fl)	-1.500<br>(1.951)<br>$p = 0.442$	-1.455*<br>(0.492)<br>$p = 0.004$	-0.364<br>(0.492)<br>$p = 0.461$	0.788<br>(0.682)<br>$p = 0.248$	1.114<br>(0.682)<br>$p = 0.103$	70.000*<br>(1.409)<br>$p < 0.001$	-146.241	308.482
MCH<br>(pg)	-0.343<br>(0.558)<br>$p = 0.539$	0.536*<br>(0.125)<br>$p < 0.001$	-0.018<br>(0.125)<br>$p = 0.885$	-0.095<br>(0.173)<br>$p = 0.585$	0.110<br>(0.173)<br>$p = 0.527$	20.118*<br>(0.403)<br>$p < 0.001$	-62.511	141.021
MCHC<br>(g/l)	1.727<br>(3.873)<br>$p = 0.656$	14.000*<br>(2.406)<br>$p < 0.001$	0.909<br>(2.406)<br>$p = 0.706$	-3.250<br>(3.330)<br>$p = 0.330$	-2.159<br>(3.330)<br>$p = 0.517$	287.273*<br>(2.797)<br>$p < 0.001$	-224.727	465.454
RDW<br>(%)	0.196<br>(0.372)<br>$p = 0.598$	0.791*<br>(0.202)<br>$p < 0.001$	-0.000<br>(0.202)<br>$p = 1.000$	-0.041<br>(0.279)<br>$p = 0.884$	-0.108<br>(0.279)<br>$p = 0.698$	21.445*<br>(0.269)<br>$p < 0.001$	-72.193	160.386
PCT<br>(%)	-0.015<br>(0.016)<br>$p = 0.331$	0.013<br>(0.011)<br>$p = 0.247$	-0.013<br>(0.011)<br>$p = 0.247$	0.003<br>(0.015)<br>$p = 0.841$	0.034*<br>(0.015)<br>$p = 0.030$	0.082*<br>(0.011)<br>$p < 0.001$	116.877	-217.754
PLT<br>(10^{9}/l)	-23.871<br>(21.226)<br>$p = 0.261$	20.636<br>(14.424)<br>$p = 0.153$	-19.727<br>(14.424)<br>$p = 0.172$	7.220<br>(20.261)<br>$p = 0.722$	42.584*<br>(20.261)<br>$p = 0.036$	128.182*<br>(15.131)<br>$p < 0.001$	-329.453	674.905
MPV<br>(fl)	0.122<br>(0.163)<br>$p = 0.454$	0.145<br>(0.111)<br>$p = 0.191$	0.145<br>(0.111)<br>$p = 0.191$	-0.278<br>(0.156)<br>$p = 0.076$	-0.228<br>(0.156)<br>$p = 0.145$	6.218*<br>(0.116)<br>$p < 0.001$	-27.737	71.475
PDW<br>(%)	0.270<br>(0.599)<br>$p = 0.653$	0.673<br>(0.448)<br>$p = 0.134$	0.136<br>(0.448)<br>$p = 0.761$	-0.975<br>(0.629)<br>$p = 0.121$	-0.064<br>(0.629)<br>$p = 0.919$	26.891*<br>(0.426)<br>$p < 0.001$	-111.124	238.248

Variable (unit)	Group M	Time TC	Time T6	Group M :Time TC	Group M :Time T6	Constant	Log Likelihood	AIC
WBC (10^9/l)	-4.169* (1.044) *p* < 0.001	-0.754 (0.704) *p* = 0.285	1.680* (0.704) *p* = 0.018	2.056* (0.975) *p* = 0.035	3.297* (0.975) *p* = 0.001	15.152* (0.754) *p* < 0.001	-144.967	305.935
BANDS% (%)	-0.477 (0.996) *p* = 0.632	1.909* (0.896) *p* = 0.034	-0.636 (0.896) *p* = 0.478	-0.992 (1.241) *p* = 0.424	-0.030 (1.241) *p* = 0.981	2.727* (0.719) *p* < 0.001	-150.054	316.108
SEG% (%)	-8.523 (4.563) *p* = 0.062	-23.818* (4.198) *p* < 0.001	14.091* (4.198) *p* = 0.001	15.152* (5.812) *p* = 0.010	14.992* (5.812) *p* = 0.010	61.273* (3.296) *p* < 0.001	-246.360	508.720
LYM% (%)	3.212 (3.119) *p* = 0.304	14.273* (2.789) *p* < 0.001	-6.545* (2.789) *p* = 0.019	-8.273* (3.861) *p* = 0.033	-5.871 (3.861) *p* = 0.129	20.455* (2.253) *p* < 0.001	-221.857	459.715
MON% (%)	1.712 (1.246) *p* = 0.170	1.636 (1.195) *p* = 0.171	-1.636 (1.195) *p* = 0.171	-3.386* (1.655) *p* = 0.041	-3.364* (1.655) *p* = 0.043	8.455* (0.900) *p* < 0.001	-165.207	346.415
EOS% (%)	4.076* (1.912) *p* = 0.034	6.000* (1.762) *p* = 0.001	-5.273* (1.762) *p* = 0.003	-2.500 (2.440) *p* = 0.306	-5.727* (2.440) *p* = 0.019	7.091* (1.381) *p* < 0.001	-191.586	399.172
NEU (10^9/l)	-3.687* (0.997) *p* < 0.001	-3.909* (0.799) *p* < 0.001	3.208* (0.799) *p* < 0.001	3.558* (1.107) *p* = 0.002	3.902* (1.107) *p* < 0.001	9.892* (0.720) *p* < 0.001	-147.390	310.779
LYM (10^9/l)	-0.540 (0.474) *p* = 0.255	2.071* (0.438) *p* < 0.001	-0.708 (0.438) *p* = 0.106	-0.953 (0.606) *p* = 0.116	0.023 (0.606) *p* = 0.970	3.012* (0.343) *p* < 0.001	-103.777	223.554
EPI (nmol/l)	-0.854 (2.446) *p* = 0.728	7.193* (1.844) *p* < 0.001	-0.631 (1.844) *p* = 0.733	-2.304 (2.552) *p* = 0.367	0.501 (2.552) *p* = 0.845	2.061 (1.767) *p* = 0.244	-202.145	420.290
Cortisol (nmol/l)	14.605 (15.400) *p* = 0.343	-55.445* (13.742) *p* < 0.001	-42.873* (13.742) *p* = 0.002	-19.621 (19.025) *p* = 0.303	17.123 (19.025) *p* = 0.369	107.645* (11.123) *p* < 0.001	-322.416	660.833
N:L ratio	-0.722 (1.971) *p* = 0.715	-2.439 (1.905) *p* = 0.201	4.768* (1.905) *p* = 0.013	1.165 (2.637) *p* = 0.659	2.035 (2.637) *p* = 0.441	3.768* (1.424) *p* = 0.009	-103.777	404.355
LCC (RLU)	953.009 (1,153.042) *p* = 0.409	-1,340.531 (1,135.837) *p* = 0.238	1,200.595 (1,135.837) *p* = 0.291	-126.896 (1,572.495) *p* = 0.936	-2,146.912 (1,572.495) *p* = 0.173	3,983.613* (832.859) *p* < 0.001	-595.769	1,207.538
FGN (g/l)	-0.133 (0.179) *p* = 0.455	0.458* (0.144) *p* = 0.002	0.115 (0.144) *p* = 0.428	0.118 (0.203) *p* = 0.560	-0.125 (0.205) *p* = 0.545	1.795* (0.127) *p* < 0.001	-37.622	91.244
HP (g/l)	0.370 (0.451) *p* = 0.413	0.215* (0.094) *p* = 0.022	0.046 (0.094) *p* = 0.622	0.100 (0.130) *p* = 0.444	-0.081 (0.132) *p* = 0.542	0.465 (0.326) *p* = 0.154	-45.848	107.697
ALB (g/l)	0.023 (0.554) *p* = 0.968	1.970* (0.392) *p* < 0.001	0.375 (0.392) *p* = 0.340	0.823 (0.543) *p* = 0.130	1.118* (0.551) *p* = 0.043	24.599* (0.400) *p* < 0.001	-105.104	226.208
iron (µmol/l)	0.008 (1.003) *p* = 0.994	4.300* (0.834) *p* < 0.001	-6.282* (0.834) *p* < 0.001	-0.908 (1.154) *p* = 0.432	1.138 (1.170) *p* = 0.331	17.309* (0.724) *p* < 0.001	-146.451	308.901
TG (mmol/l)	0.004 (0.051) *p* = 0.931	-0.105* (0.046) *p* = 0.021	-0.192* (0.046) *p* < 0.001	0.009 (0.063) *p* = 0.890	0.053 (0.064) *p* = 0.410	0.466* (0.037) *p* < 0.001	36.582	-57.165

Variable (unit)	Group M	Time TC	Time T6	Group M :Time TC	Group M :Time T6	Constant	Log Likelihood	AIC
PL (mmol/l)	-0.029 (0.022) *p* = 0.189	-0.047* (0.021) *p* =0.028	-0.004 (0.021) *p* = 0.866	0.031 (0.030) *p* = 0.303	0.002 (0.030) *p* = 0.946	0.159* (0.016) *p* < 0.001	87.187	-158.374
CD (µmol/l)	0.023 (0.040) *p* = 0.558	0.074* (0.025) *p* = 0.004	0.061* (0.025) *p* = 0.014	-0.013 (0.035) *p* = 0.709	0.016 (0.035) *p* = 0.642	0.130* (0.029) *p* < 0.001	62.182	-108.364
TBARS (µmol/l)	0.417 (0.390) *p* = 0.286	0.0003 (0.354) *p* = 1.000	0.310 (0.354) *p* = 0.381	0.090 (0.490) *p* = 0.855	-0.524 (0.496) *p* = 0.291	0.001 (0.282) *p* = 0.997	-89.834	195.668
ORAC (µmol/l)	270.500 (176.595) *p* = 0.126	289.818* (113.537) *p* = 0.011	185.909 (113.537) *p* = 0.102	-358.402* (157.185) *p* = 0.023	-338.482* (159.519) *p* = 0.034	1,104.000* (127.557) *p* < 0.001	-459.373	223.554
